# Prevalence of multiple drug resistant *Streptococcus suis* in and around Guwahati, India

**DOI:** 10.14202/vetworld.2017.556-561

**Published:** 2017-05-28

**Authors:** Mrinalee Devi, Jyoti B. Dutta, Swaraj Rajkhowa, Dhireswar Kalita, Girindra Kumar Saikia, Bipin Chandra Das, Razibuddin Ahmed Hazarika, Gauranga Mahato

**Affiliations:** 1 Department of Veterinary Epidemiology and Preventive Veterinary Medicine, College of Veterinary Science, AAU, Khanapara, Guwahati, Assam - 781 022, India; 2 National Research Centre on Pig, ICAR, Rani, Kamrup, Assam - 781 131, India; 3 AICRP/MSP on Pigs, College of Veterinary Science, AAU, Khanapara, Guwahati, Assam - 781 022, India; 4 Department of Veterinary Microbiology, College of Veterinary Science, AAU, Khanapara, Guwahati, Assam - 781 022, India; 5 Department of Veterinary Public Health, College of Veterinary Science, AAU, Khanapara, Guwahati, Assam - 781 022, India

**Keywords:** antimicrobial resistance, emerging zoonotic pathogen, multiple drug resistance, pathogen prevalence, *Streptococcus suis*

## Abstract

**Aim:**

This study was conducted to determine the prevalence and antimicrobial susceptibility of *Streptococcus suis* and their resistance patterns isolated from both clinically healthy carriers and diseased pigs in and around Guwahati, Assam, India.

**Materials and Methods:**

A total of 497 samples were collected during October, 2012, to April, 2014, from clinically healthy (n=67) and diseased (n=230) pigs of varying age and either sex maintained under organized and unorganized farming systems. Samples were processed for isolation and identification of *S. suis* by biochemical characterization and polymerase chain reaction targeting the housekeeping gene glutamate dehydrogenase. *In vitro* antimicrobial susceptibility of the recovered isolates against nine antibiotic groups comprising 17 antimicrobial agents was studied by standard method.

**Results:**

Of the 497 samples examined, 7 (1.41%) isolates were confirmed to be *S. suis* of which 5 (1.87%) and 2 (0.87%) were derived from clinically healthy and diseased pigs, respectively. All the isolates were susceptible to gentamicin, amikacin, and erythromycin (100%) followed by the penicillin group and enrofloxacin (85.71%), ceftriaxone, doxycycline HCL, ofloxacin and chloramphenicol (71.43%), to kanamycin, clindamycin and co-trimoxazole (42.85%). The isolates showed least susceptibility to cefalexin, tetracycline and streptomycin (28.57%). All the five *S. suis* isolates from clinically healthy pigs were susceptible to penicillin G, amoxyclav, doxycycline HCl, gentamicin, amikacin and erythromycin, 80.00% isolates susceptible to ampicillin, enrofloxacin and ofloxacin, 60.00% to ceftriaxone, kanamycin and chloramphenicol, 40% to cefalexin, tetracycline, clindamycin and co-trimoxazole, respectively. Only 20.00% isolates were susceptible to streptomycin. Both the isolates recovered from diseased pigs were susceptible to ampicillin, ceftriaxone, gentamicin, amikacin, enrofloxacin, erythromycin, and clindamycin. On the other hand, both the isolates were resistant to cefalexin, tetracycline, doxycycline HCL, and kanamycin. Altogether five different resistance patterns (multi-drug resistance) were observed. Of the seven *S. suis* isolates, two isolates were susceptible to all the 17 antimicrobial agents, one isolate was resistant to four antimicrobial agents, two isolates to seven agents, one isolate to nine agents, and one isolate exhibited resistance to 14 antimicrobial agents.

**Conclusion:**

This study was conducted to determine the prevalence of *S. suis* in clinically healthy and diseased pigs and their antimicrobial susceptibility patterns. All the isolates were susceptible to gentamicin, amikacin and erythromycin, and most of them were resistant to cefalexin, tetracycline and streptomycin. Five different patterns of antimicrobial resistance (multi-drug resistance) were observed.

## Introduction

*Streptococcus suis* is recognized worldwide as one of the most important and prevalent swine pathogens mostly associated with meningitis, septicemia, endocarditis, arthritis, pneumonia, occasionally, polyserositis, pericarditis, endometritis, abortion, rhinitis, and vaginitis leading to high mortality and considerable economic losses [1-4]. At present, there are 35 recognized serotypes of *S. suis* based on capsular polysaccharides, of which serotype 2 is highly prevalent and virulent and most frequently isolated from diseased pigs and human [3,5]. *S. suis* 2 is also recognized as one of the most important emerging zoonotic pathogens and has been isolated from a wide range of mammalian species including humans and humans are often infected via skin wounds during contact with pigs and their products [1,6,7]. Slaughter-house pigs were reported to be major reservoir of *S. suis* [8].

Owing to the antigenic variations, currently no effective vaccines against *S. suis* are available [9]. Current vaccines are bacterins providing partial serotype-specific protection [10]. In the absence of effective vaccines antimicrobial agents are increasingly used in treating and controlling *S. suis* infection. Susceptibilities of strains from diseased and clinically healthy pigs in different countries were investigated [5,9,11] of which *β*-lactams, tetracyclines, sulfonamides, and macrolides were the frequently used antimicrobials for prevention and treatment of streptococcal infections in pigs. Variations in the level of antimicrobial resistance were observed among different countries, serotypes, and over time [11]. *S. suis* isolated from infected or clinically healthy pigs were often resistant to commonly used antimicrobial agents [5,9,11]. Indiscriminate use of antibiotics has led to the development of resistance of *S. suis* to these drugs worldwide [5,9-11].

The study on prevalence and antimicrobial susceptibility patterns of *S. suis* recovered from clinically healthy as well as diseased pigs are important factors for controlling outbreaks. However, such information is limited to endemic areas. The objectives of this study were to determine the prevalence of *S. suis* and antimicrobial susceptibility as well as multi-drug resistance patterns of the isolates from clinically healthy and diseased pigs.

## Materials and Methods

### Ethical approval

The research work was duly permitted by the Institutional Animal Ethics Committee. All samples were collected as per standard procedure without harming or laying stress to the animals.

### Sample collection and processing

The study was conducted during October 2012 to April 2014. A total of 497 samples comprising clinically healthy (n=267) and diseased pigs (n=230) of various age groups of either sex were collected from both organized (n=2; AICRP/MSP on Pig, College of Veterinary Science, Assam Agricultural University, Khanapara, Guwahati - 781 022 and National Research Center on Pig, Rani, Kamrup - 781 131, Assam) and unorganized farms (n=51; private pig farms located around Guwahati, viz., Changchari, Tangla and Bonda). Samples (nasal swabs, joint fluid, heart blood from live pigs and heart fluid, and pieces of lungs from dead pigs) were collected aseptically following standard procedure from clinically healthy pigs as well as pigs exhibiting respiratory tract involvement and died of respiratory disease. The nasal samples were collected with the help of sterile cotton-tipped applicator sticks (Hiculture transport swab, HiMedia Laboratories Pvt. Ltd., Mumbai - 400 086). All the samples were transported to the laboratory at 4-8°C in ice-boxes within 24-48 h of collection for further processing.

### Bacteria isolation and identification

Samples were directly inoculated into Todd Hewitt broth (HiMedia Laboratories Pvt. Ltd., Mumbai - 400 086) and incubated overnight at 37°C aerobically. Subsequently, isolates were grown on nutrient agar (NA) plates to obtained pure colonies. The isolates were screened for hemolytic reaction on NA plates supplemented with 5% sheep blood and subjected for different conventional biochemical tests [12]. Isolates were provisionally identified as *S. suis* when they were non beta-hemolytic, negative for the Voges-Proskauer tests, positive for esculin and hippurate hydrolysis, produced acid from trehalose but did not produce from sorbitol and mannose. There was no growth on agar supplemented with 6.5% NaCl. The isolates were finally confirmed by polymerase chain reaction specific for glutamate dehydrogenase gene [13].

### Antimicrobial susceptibility test

*In vitro* antimicrobial susceptibility of isolated bacteria were tested by the disc diffusion technique according to CLSI [14] using Mueller-Hinton agar (HiMedia) medium against nine antimicrobial groups comprising 17 agents (Table-1).

**Table-1:** Groups of antimicrobial agents and their concentrations.

S.No.	Antimicrobial group	Agents	Symbols	Concentration per disc
1	Penicillin	Penicillin-G	P	10 IU
		Ampicillin	AMP	10 mcg
		Amoxyclav	AMC	30 mcg
2	Cephalosporin	Cefalexin	CN	30 mcg
		Ceftriaxone	CTR	30 mcg
3	Tetracycline	Tetracycline	TE	30 mcg
		Doxycycline HCl	DO	30 mcg
4	Aminoglycoside	Streptomycin	HLS	300 mcg
		Gentamicin	HLG	120 mcg
		Kanamycin	K	30 mcg
		Amikacin	AK	30 mcg
5	Fluoroquinolone	Enrofloxacin	EX	10 mcg
		Ofloxacin	OF	5 mcg
6	Macrolide	Erythromycin	E	15 mcg
7	Lincosamide	Clindamycin	CD	2 mcg
8	Chloramphenicol	Chloramphenicol	C	30 mcg
9	Sulfa group	Co-trimoxazole (sulfamethoxazole - trimethoprim)	COT	25 mcg

## Results

### Prevalence of S. suis

In this study, seven *S. suis* (1.41%) isolates were obtained from 497 samples of which 5 (1.87%) and 2 (0.87%) were derived from clinically healthy and diseased pigs, respectively.

### Antimicrobial susceptibility of S. suis

It was observed that all the isolates were susceptible to gentamicin, amikacin and erythromycin (100%) followed by the penicillin group and enrofloxacin (6 isolates, 85.71%), ceftriaxone, doxycycline HCL, ofloxacin and chloramphenicol (5 isolates, 71.43%), kanamycin, clindamycin, and co-trimoxazole (3 isolates, 42.85%). Cefalexin, tetracycline, and streptomycin (2 isolates, 28.57%) were the least effective agents (Table 2).

**Table-2:** Antimicrobial susceptibility of *S. suis*.

S.No.	Antimicrobial group	Agents	*S. suis*		

			No. of susceptible isolates (n=7)	Clinically healthy pigs (n=5)	Diseased pigs (n=2)
1	Penicillin group	P	6 (85.71)*	5 (100.00)	1 (50.00)
		AMP	6 (85.71)	4 (80.00)	2 (100.00)
		AMC	6 (85.71)	5 (100.00)	1 (50.00)
2	Cephalosporins	CN	2 (28.57)	2 (40.00)	0
		CTR	5 (71.43)	3 (60.00)	2 (100.00)
3	Tetracyclines	TE	2 (28.57)	2 (40.00)	0
		DO	5 (71.43)	5 (100.00)	0
4	Aminoglycosides	HLS	2 (28.57)	1 (20.00)	1 (50.00)
		HLG	7 (100.00)	5 (100.00)	2 (100.00)
		K	3 (42.85)	3 (60.00)	0
		AK	7 (100.00)	5 (100.00)	2 (100.00)
5	Fluoroquinolones	EX	6 (85.71)	4 (80.00)	2 (100.00)
		OF	5 (71.43)	4 (80.00)	1 (50.00)
6	Macrolide	E	7 (100.00)	5 (100.00)	2 (100.00)
7	Lincosamide	CD	3 (42.85)	2 (40.00)	1 (50.00)
8	Chloramphenicol	C	5 (71.43)	3 (60.00)	2 (100.00)
9	Sulfa-drug	COT	3 (42.85)	2 (40.00)	1 (50.00)

* Figures in parentheses indicate percentages.*S. suis=Streptococcus suis*

The antibiogram of *S. suis* isolates (n=5) from clinically healthy pigs showed that all the isolates susceptible to penicillin G and amoxyclav (100%), whereas only 4 (80%) isolates were susceptible to ampicillin in the penicillin group. In the cephalosporin group, 40% (n=2) and 60% (n=3) isolates were susceptible to cefalexin and ceftriaxone, respectively. Among the tetracyclines, 100% susceptibility to doxycycline HCl was observed, but only 40% isolates (n=2) were susceptible to tetracycline. In case of aminoglycosides, 100% susceptibility to gentamicin and amikacin was observed but 60.00% isolates (3 isolates) were susceptible to kanamycin. 80% isolates (4 isolates) were susceptible to fluoroquinolones, i.e., enrofloxacin and ofloxacin. All the isolates were susceptible to macrolides, i.e., erythromycin (100%). The isolates were moderately susceptible to chloramphenicol (60%), and only 40% (n=2) were susceptible to clindamycin and sulfa group (Table-2).

The antibiogram of the two isolates recovered from diseased pigs showed that both the isolates were susceptible (100%) to ampicillin, ceftriaxone, gentamicin, amikacin, enrofloxacin, erythromycin, and clindamycin. On the other hand, these isolates showed 100% resistance to cefalexin, tetracycline, doxycycline HCL, and kanamycin.

### Antimicrobial resistance pattern(s) of *S. suis*

Altogether five different resistance patterns were observed in this study and described in Table-3.

**Table-3:** Antimicrobial resistance pattern (s) of *Streptococcus suis*.

S.No	No. of isolates (n=7)	Antimicrobials tested (n=17)			

		Sensitive		Resistant	
	
		Susceptible to	No. of isolates	Resistant to	No. of isolates
1	2	P, AMP, AMC, CN, CTR, TE, DO, HLS, HLG, K, AK, EX, OF, E, CD, C, COT	17	-	0
2	1	P, AMP, AMC, CTR, DO, HLG, K, AK, EX, E, CD, C, COT	13	CN, TE, HLS, OF	4
3	2	P, AMP, AMC, CTR, DO, HLG, AK, EX, E, C,	10	CN, TE, HLS, K, CD, COT, OF	7
4	1	P, AMP, AMC, CTR, HLG, AK, EX, E	8	CN, TE, HLS, K, OF, CD, C, DO, COT	9
5	1	HLG, AK, E,	3	P, AMP, AMC, CN, CTR, TE, DO, HLS, K, EX, OF, CD, C, COT	14

## Discussion

### Prevalence of S. suis

In this study, *S. suis* were isolated from both clinically healthy and diseased pigs where higher prevalence was recorded in clinically healthy pigs. Several workers had reported *S. suis* strains from clinically healthy pigs [15-18] as well as from various diseased conditions such as septicemia, meningitis, arthritis, and fibrinous polyserositis; with bronchopneumonia [17-19] and endocarditis [20]. The findings of the present study are in agreement with Padungtod *et al*. [21] reporting a higher incidence of *S. suis* in clinically healthy pigs (43%) than in diseased pigs (10%) indicating a key role of clinically healthy carriers in spreading the infection to other pigs as well as human.

Comparing the reports of incidence, a low incidence of *S. suis* in clinically healthy and diseased pigs was recorded indicating a lower prevalence of this pathogen in this locality. Low prevalence was also reported earlier [17] in similar locality.

### Antimicrobial susceptibility of S. suis

In the present study, all the isolates were highly susceptible to gentamicin, amikacin, and erythromycin followed by the penicillin group and enrofloxacin. A proportionately higher susceptibility to *β*-lactams and aminoglycosides were also reported previously [5,11,17,22]. On the contrary, high levels of resistance to these agents were also reported by various workers [9,19,23-25].

The antibiogram of the isolates had showed some differences with previous reports in the present study. A high frequency of resistance to tetracyclines, sulfonamides, macrolides, chloramphenicol, kanamycin, clindamycin, and co-trimoxazole was demonstrated by several workers [10,15,17,23,24,26], but the present isolates were highly susceptible to macrolides and chloramphenicol. The higher percentages of resistance to these agents could be related to the widespread use of these antimicrobials in veterinary practice. In Italy and other countries of Europe, the rising rates of resistance have been attributed to extensive use of Tylosin, a macrolides class of antibiotic as a growth promoter and of tetracycline as a therapeutic agent [6]. Tetracycline resistance in *S. suis* isolated from pig in various locations of Asia and Europe were reported by many workers [9,17,22,26] significantly increasing over time [8]. Several reports demonstrated high level of susceptibility to cefalexin and ceftriaxone [5,10], but the present study revealed a high level of resistance to these two agents along with streptomycin which is in agreement with previous reports [17,22]. The findings indicate an increased resistance to the commonly used antimicrobial agents reflecting regional variations.

### Antimicrobial resistance pattern(s) of *S. suis*

Five different resistance patterns were observed in the present study. Many workers had reported resistance of *S. suis* to multiple antimicrobial agents as high as 100% [27]. Resistance to at least four antimicrobial agents in 87% of the isolates and 6% to at least 6 antimicrobials were reported [11] where the most frequently identified multi-drug resistance patterns involved resistance against tetracyclines, sulfonamides, macrolides and lincosamides (69%). In a 42-year study [8], it was observed that multi-drug resistance (resistance to tetracycline, erythromycin and chloramphenicol) of the *S. suis* isolated from adult human patients significantly increased over time. Isolates of *S. suis* from diseased pigs in Southwest China exhibited multiple drug resistance against ampicillin, amoxicillin, amoxicillin/clavulanate, clindamycin, amikacin, gentamicin, erythromycin, and tetracycline [19]. Multi-drug resistant of *S. suis* isolated from clinically healthy pigs in Brazil [10] showed different resistance patterns, viz. resistance to at least to 3 antimicrobials (99.61%), at least to 4 antimicrobials (97.30%), at least to 6 (85%) antimicrobials, and 9 strains were resistant to all the antimicrobial tested.

## Conclusion

This study was conducted to determine the prevalence of *S. suis* in clinically healthy and diseased pigs of all age groups and either sex in and around Guwahati reared under organized and unorganized system of farming. The antimicrobial resistance patterns exhibited by the isolates were also observed. A higher percentage of *S. suis* isolated from clinically healthy pigs indicated a carrier status with risk of dissemination to other pigs in the herd as well as to humans. On susceptibility testing to 17 antimicrobials of nine major groups, only 28.57% isolates had showed 100% susceptibility. The study revealed five different antimicrobial resistance patterns showing resistance to maximum number of antimicrobials. This finding not only poses a serious threat to the pig industry leaving few effective therapeutic agents but also has zoonotic perspective causing morbidity in pig handlers as well as pork processors and consumers alike. These resistance carrier isolates may also be incriminated for the development of drug resistance in human isolates leading to complications in antimicrobial therapy.

## Authors’ Contributions

MD carried out the experiment and collected the scientific literatures and prepared the first draft of the manuscript. JBD guided in drafting the final manuscript. JBD and SW designed the experiment and guided during the research work. DK provided necessary facilities for collection of samples from pig farm. GKS, BCD, GM, and RAH provided their valuable advice during the whole experiment. All the authors read and approved the final manuscript.
